# Single Low-Dose Methotrexate and Vitamin B12 Deficiency-Induced Pancytopenia Causing Fatality: A Case Report

**DOI:** 10.7759/cureus.63528

**Published:** 2024-06-30

**Authors:** Ghida Akhdar, Inemesit Akpan, Amanda Myles, Stanley E Atencah

**Affiliations:** 1 Internal Medicine, Piedmont Athens Regional Medical Center, Athens, USA; 2 Anesthesiology, Boston University School of Medicine, Boston, USA

**Keywords:** vitamin b12 deficiency, methotrexate-induced pancytopenia, methotrexate, refractory pancytopenia, fatality

## Abstract

Methotrexate (MTX), a commonly used disease-modifying antirheumatic drug, is generally considered safe at low cumulative doses. However, severe pancytopenia can occur even with doses as low as 10 mg, as illustrated by a fatal case in an older adult with chronic kidney disease (CKI) and vitamin B12 deficiency. Despite the low dose and lack of folate supplementation, the patient developed severe mucositis and pancytopenia leading to fatal complications. This case underscores the challenges in diagnosing and managing MTX-induced pancytopenia, especially in patients with comorbidities such as CKI and vitamin B12 deficiency.

## Introduction

Methotrexate (MTX) hematological toxicity, including severe pancytopenia, is underreported, with few fatal cases described. MTX, widely used in rheumatological diseases, is considered safe, with 3% rates of hematological toxicity and 1% to 1.4% of pancytopenia [1,2]. Most cases are in rheumatoid arthritis treatment. Here, we present a case of pancytopenia and mucositis after just 10 mg MTX, complicated by B12 deficiency. Identifying whether MTX alone, B12 deficiency alone, or both caused pancytopenia was challenging. Despite appropriate management, the lack of clinical response complicated the diagnosis. Bone marrow findings were inconclusive, lacking guidance for interpretation.

## Case presentation

A 71-year-old patient, with chronic kidney disease (CKI) and psoriatic arthritis, initiated two weeks ago on MTX 5 mg weekly, presented with severe mucositis, bleeding mouth ulcers, fever, and generalized weakness. 

Upon presentation, lab work exhibited pancytopenia, with a hemoglobin of 7.1 g/dL, macrocytosis (MCV: 108 fL), and white blood cell count of 1.6x10^3^/μL with initial mild neutropenia (absolute neutrophil count: 1009), lymphopenia, and thrombocytopenia (platelets: 31000/μL) (Table 1). Investigations unveiled low vitamin B12 level (94 pg/mL) and low normal folate. He had acute kidney injury (AKI) on top of his CKI with a creatinine of 1.9 compared to 1.3 as a baseline. Transaminases were normal but alkaline phosphatase was mildly elevated with an increase of both direct and indirect bilirubin and no hypoalbuminemia. Peripheral smear revealed macrocytosis, reticulocytopenia, and rare hypersegmented neutrophils with very rare schistocytes. 

**Table 1:** Laboratory values on presentation

Laboratory test	Value	Reference range
Hemoglobin	7.1 g/dL	Male: 13.5-17.5 g/dL; female: 12.0-16.0 g/dL
MCV	108 fL	80-100 fL
WBC	1.6	4.5-11.0×109/L
Absolute neutrophils count	1009	2500-7000 cells/mL
Platelets	31	150-400×109/L
Creatinine	1.9	0.6-1.2 mg/dL
Calcium	7.8	8.4-10.2 mg/dL
ALT	10	10-40 U/L
AST	15	12-38 U/L
ALKP	202	25-100 U/L
T bili	1.6	0.1-1.0 mg/dL
INR	1.4	0.8-1.1
Albumin	1.2	3.5-5.5 g/dL
Vitamin B12	94 pg/mL	160-950 pg/mL
Folate	4	2.7-17.0 ng/mL

The patient's hospital course involved consultations from hematology, gastroenterology, and infectious diseases. Testing for HIV, hepatitis, acute CMV, parvovirus, EBV, and HSV returned negative. Due to painful oral ulcers concerning for HSV or CMV, he received empirical Ganciclovir, but then discontinued after negative results.

Flow cytometry was inconclusive, revealing an expansion of T-large granular lymphocytes without evidence of neoplastic populations. Cytogenetics was done but no metaphase cells were detected to obtain results, likely related to very low cellularity. Bone marrow biopsy revealed hypocellular <5%, trilineage hypoplasia and no blasts. Stainable iron was present. FISH studies were negative for monosomy/deletion of chromosomes 5, 7, 13, 17, and 20, trisomy 8, and for MECOM and MT2A rearrangements.

Throughout the hospitalization, the patient received seven days of daily intramuscular 1000 mcg B12 injections alongside oral 1000 mcg B12 supplementation. He required frequent transfusions without response and no evidence of gastrointestinal bleeding on endoscopy. Despite G-CSF, minimal response was observed. After 10 days and persistent severe neutropenia, our patient went into acute hypoxic respiratory failure thought to be related to fluid overload with superimposed hospital-acquired pneumonia. Discussions on goals of care transpired, with inpatient hospice transfer, where he eventually succumbed to his complex medical condition.

## Discussion

MTX is a folate antagonist widely utilized in the management of autoimmune disorders including psoriatic arthritis. Although MTX-induced pancytopenia is reportedly rare with only 1.4% of the cases, fatality with low cumulative doses <25 mg is even rarer [1,3]. Pancytopenia resulting from a low cumulative dose of MTX of less than 25 mg was only reported in six cases [1,2,4-6]. The scarcity of reported cases underscores the clinical significance of MTX-induced pancytopenia, which can mislead a clinical judgment when facing such a scenario. A study by Vyas et al. presented three cases where patients developed pancytopenia following MTX therapy for various indications, emphasizing the diversity of presentations [7]. Similarly, Haroon et al. reported 25 cases of MTX-induced pancytopenia seen within the span of five years, shedding light on the possible severity of it [7]. Risk factors play a major role in the intensity of MTX hematological toxicity. Renal impairment can tremendously potentiate MTX toxicity, which was the case with our patient. Dose adjustments and very close monitoring are crucial among patients with known renal impairment [8]. Another risk factor is the lack of folate supplementation or patients’ medical illiteracy that leads to errors in dosing. Genetic polymorphism in the MTHFR gene (C677T and 1298AA) can also predispose to higher risk side effects with the use of MTX, but no routine testing is done nor recommended. Our patient also had notable vitamin B12 deficiency. The literature review did not demonstrate an established role of vitamin B12 deficiency in potentiation of MTX toxicity. In fact, it is not yet clear how low-dose MTX works. A suggestion might be that MTX inhibits methyl donation [9]. MTX acts as a dihydrofolate reductase antagonist, reducing S-adenosylmethionine (SAM), the main methyl donor. Vitamin B12 deficiency increases homocysteine, reducing SAM synthesis. MTX and B12 deficiency theoretically lower SAM levels, though not confirmed. Our patient lacked folate supplementation and had CKI and B12 deficiency, which can exacerbate MTX toxicity. Reduced RBC folate, seen with MTX-induced pancytopenia and macrocytosis, suggested B12 deficiency. AKI on CKD might worsen MTX toxicity or result from volume depletion due to mucositis. Amid worsening pancytopenia, a bone marrow biopsy was warranted due to multiple potential contributors. The results were concordant with previously reported findings of bone marrow biopsies found with MTX toxicity in the literature: hypocellular marrow with trilineage hypoplasia consistent with myelosuppression induced by MTX [10]. There were also reported neutrophils hypersegmentation and erythrocyte budding, a finding that is also found in B12 deficiency. The absence of clonality and/or blasts differentiates it from MDS. Our patient ended up unfortunately dying, which is extremely rare or underreported with low cumulative doses. This raises the concern about a possible underlying idiosyncratic reaction. In a study conducted by Mori et al., 40 patients treated with a relatively low dose of MTX were retrospectively analyzed and concluded that MTX-induced pancytopenia can happen at any point and severe neutropenia with ANC <500 was more prevalent during the first two months of treatment [11]. Moreover, another Scandinavian study concluded that MTX hematological toxicity, specifically pancytopenia, is not dose-dependent, which opposes previous beliefs and literature data [12]. One helpful indicator for survival in the cases of MTX-induced pancytopenia is WBC on admission. In patients with WBC <1000, only one-third survived [12]. Other risk factors are hypoalbuminemia, advanced age, prior history of MTX toxicity, and polypharmacy with the use of more than five drugs [13]. Usually, it is expected for cytopenia to recover in the cases of MTX in five to six days duration [4,12]. Unfortunately, the longer the cytopenia persists, the higher the risk for infections and sepsis. Sepsis is the most common cause of fatality in MTX-induced cytopenia, and when looking at etiology sites, pulmonary is the number one culprit, followed by urinary and gastrointestinal. Like this case, another case was reported [6]. Unfortunately, our patient never recovered from neutropenia.

## Conclusions

MTX-induced pancytopenia is likely underreported especially when it comes to low cumulative dosage inducing severe pancytopenia and fatality. An underlying idiosyncratic reaction cannot be ruled out, which prompts very close monitoring for hematological labs in patients started on MTX, especially with concomitant risk factors. It is reasonable to test for B12 deficiency as this could constitute a double hit to the bone marrow and prolong recovery from cytopenia. 
